# Cranio-spinal migration of a metallic clip placed during arteriovenous malformation resection - A case report, review of the literature, and management strategies

**DOI:** 10.1186/1471-2377-10-109

**Published:** 2010-11-03

**Authors:** Clark C Chen, Pascal O Zinn, Ekkehard M Kasper, Christopher S Ogilvy

**Affiliations:** 1 Department of Neurosurgery, Harvard Medical School, Beth Israel Deaconess Medical Center, Boston MA 02115, USA; 2 Department of Radiation Oncology, Dana Farber Cancer Institute, Boston MA 02115, USA; 3 Department of Neurosurgery, Harvard Medical School, Massachusetts General Hospital, Boston MA 02114, USA

## Abstract

**Background:**

Microclip placement during AVM resection is generally accepted to be a safe practice in neurosurgery. Here, we describe an unusual complication involving cranio-spinal clip migration discovered five years after the initial AVM surgery.

**Case Presentation:**

A 53-year-old man underwent resection of a superior vermian AVM that required the placement of two microclips during the procedure. Five years after surgery, the patient suffered from descending sensory radiculopathy that resolved spontaneously. The workup revealed cranio-spinal migration of one of the previously placed microclips.

**Conclusions:**

AVM clip migration is a rare phenomenon; however, the diagnosis should be entertained in patients with posterior fossa instrumentation who suffer from unusual neurologic symptoms.

## Background

Feeding arteries and draining veins encountered during arteriovenous malformation (AVM) resection are often too fragile to treat by electrocautery and require microclip placement for reliable hemostasis [1]. In general, microclip placement during AVM resection is accepted as a safe practice in neurosurgery. Here, we describe a complication involving cranio-spinal clip migration discovered five years after the initial AVM surgery. The patient was managed expectantly with close clinical follow-up and remained symptom-free nine years after the initial AVM surgery. The literature and management strategies for cranio-spinal foreign object migration are reviewed.

## Case Presentation

**Figure 1:**
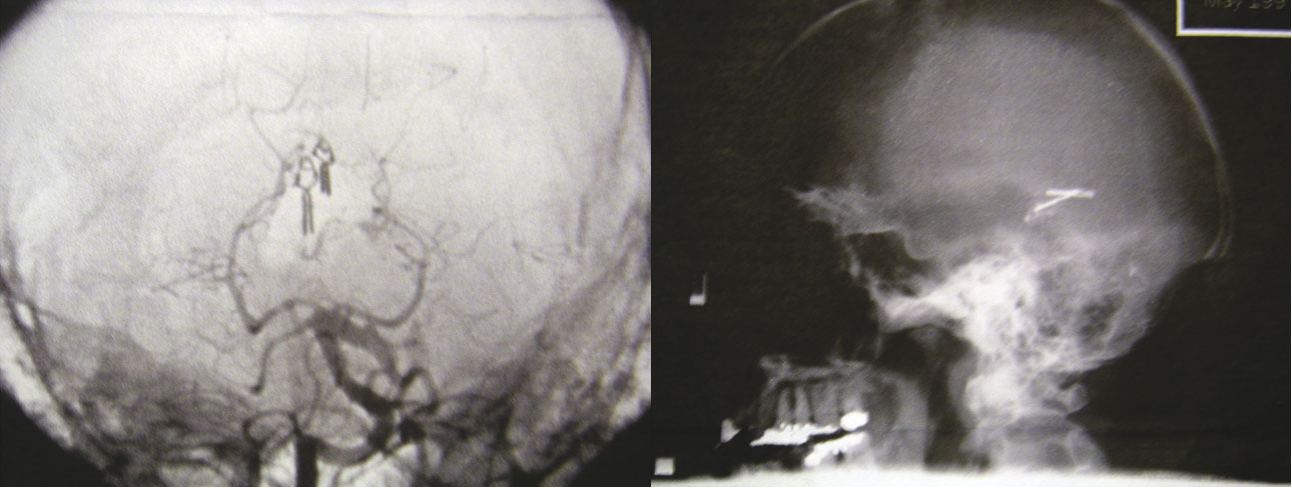
**Immediate postoperative imaging after AVM resection**: **a)** Post-operative angiogram demonstrating complete resection of the vermian AVM and **b) **lateral skull film demonstrating the initial position of the aneurysm clips.

**Figure 2:**
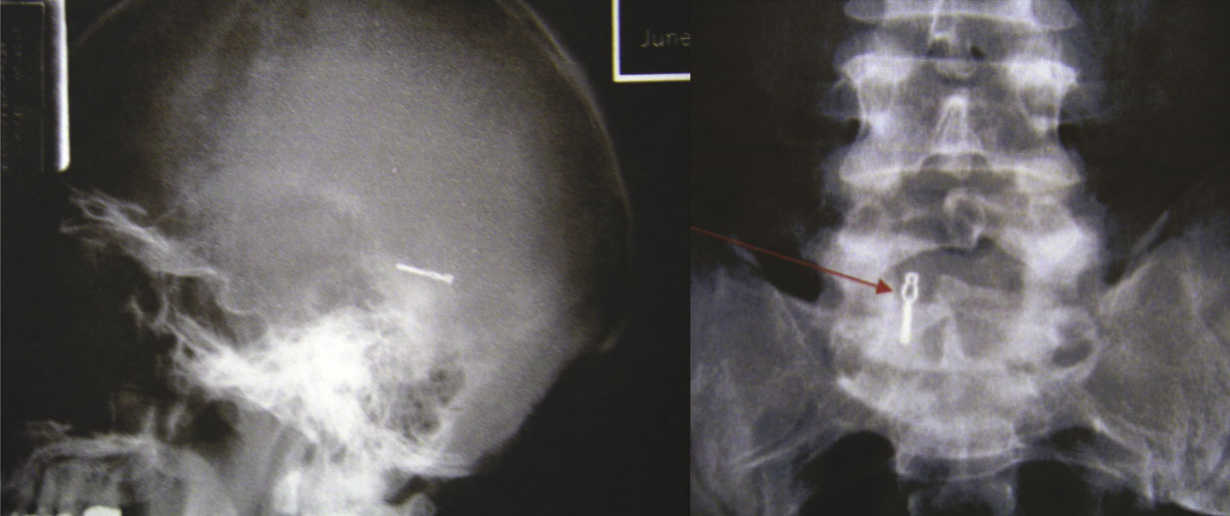
**Imaging performed five years after AVM resection**: **a)** Lateral skull film performed five years after surgery demonstrating displacement of one aneurysm clip and the absence of the other. **b) **Anterior posterior radiography of the lumbar spine revealing the location of the aneurysm clip in the lumbar spine (arrow).

A 53-year-old man underwent successful resection of a superior vermian AVM supplied by superior cerebellar arteries and drained by a single large precentral cerebellar vein. During the final stages of the surgery, two permanent Sugita aneurysm clips (Codman, Raynham, MA) were used to divide the large draining vein (Figure 1a and 1b). Post-operative angiogram revealed complete resection of the AVM. The patient remained neurologically intact after the surgery. Five years after the surgery, the patient developed neck pain that radiated to his right triceps muscle, continuing to the second and third fingers while high speed boating. The initial symptom resolved but was followed by complaints suggestive of right-sided, sequentially descending sensory radiculopathies. Each sensory level was self-resolving. The last sensory level was localized to the level of L5. Subsequent workup included a skull and lumbar spine radiography. The radiography was unremarkable in terms of fractures or dislocations. However, the skull film revealed slight inferior displacement of one of the microclips (Figure 2a). The lumbar film revealed migration of the second clip into the spinal region to the level L5 (Figure 2b). The various affected sensory levels were managed expectantly with close clinical follow-up. The patient remained symptom-free four years after the incident.

## Discussion and Conclusions

Foreign body migration in the cranial or spinal axis is a rare but established clinical entity. Most cases involve explosive splinters or retained bullets [2-6]. Migration of surgical instruments (detached sublaminar threads and cervical mass screws), epidural catheter tips, acupuncture needles, and sewing needles have also been reported [7-11]. It appears that migration can be observed with any object unattached to anatomic structures. Migration is generally detected as a result of mass effect on pain sensitive structures, infection, or incidental radiologic examinations. The delay between the initial injury/instrumentation and the discovery of migration can range between hours and years. At one end of this spectrum, a case was reported where a bullet in the left cerebellar region migrated to the contralateral hemisphere within two hours of the initial injury [3]. On the other extreme, an acupuncture needle that migrated from the posterior cervical subcutaneous tissue to C2 vertebrae over a span of 30 years was described [9].

The path of migration may not respect existing anatomic planes, especially for sharp objects. For instance, a swallowed sewing needle that migrated to the L3 vertebral body [10]. In most cases, however, natural anatomic boundaries are respected. Moreover, the path of migration can be in part, if not entirely, attributed to gravity. In the case of foreign bodies in the posterior cranial fossa, because of its anatomic continuity with the spinal canal and because of gravitational pull, migration of foreign objects into the spinal canal is expected. To date, three such cases have been reported: The migration of an intact bullet in the cisterna magna to the subarachnoid space at the C3/4 level causing right-sided C4 radiculopathy [2]. A similar case where movement of a bullet within the cisterns of the posterior fossa culminated in migration into the subarachnoid space of the spinal canal [4]. The third case involves an intracranial bullet that migrated to the cervical spinal canal over a span of four years [6].

In the case presented here, we describe the migration of a microclip that was placed during the resection of a superior vermian AVM. One of these clips migrated from the cerebellum to the L5 lumbar space over a span of five years. This clip likely became mobile as the vessel on which the clip was attached underwent necrosis in conjunction with repeated axial spine trauma resulting from high speed boating. Given the clinical context and the likely path of migration, an attractive hypothesis is that the patient's various transient symptoms of descending sensory radiculopathy resulted from temporary clip irritation of the respective nerve roots during the migration. Consistent with this hypothesis, the patient experienced permanent resolution of the symptoms without intervention.

In general, for management of symptomatic cranio-spinal foreign object migration, removal of the object is the treatment of choice for symptomatic patients [12]. An argument can be made for object removal in an otherwise asymptomatic patient in an effort to eliminate delayed consequences. However, considerations with regard to surgical morbidity and patient preference must factor into the final decision. In rare instances, migration of foreign objects is associated with abscess formation [13]. While abscess formation is less likely after sterile placement of a surgical clip, it is, nevertheless, a possibility and should be assessed. In our case, MRI and CT of the cranio-spinal axis did not reveal any evidence of abscess, infection, or clip migration-associated injury. Since the patient's symptoms were self-resolving and there were no signs of abscess/infection, he was managed expectantly with close clinical monitoring. The patient remained symptom free nine years after the initial surgery.

In conclusion, we anticipate the incidence of clip migration after neurosurgery to be a rare phenomenon. The time interval between the initial microclip placement and symptomatic onset may range months to years. The diagnosis should be entertained in neurosurgery patients who suffer from unusual neurologic symptoms, as presented here.

## Consent

This study was conducted according to HIPAA/IRB guidelines by the Harvard Medical School, MGH, and Beth Israel Deaconess Medical Center.

## Competing interests

The authors declare that they have no competing interests.

## Authors' contributions

All authors have read and approved the manuscript. CSO performed the surgery. CCC, POZ, EMK, and CSO authored the manuscript

## Pre-publication history

The pre-publication history for this paper can be accessed here:

http://www.biomedcentral.com/1471-2377/10/109/prepub
